# Supplementary material for: The PM6-FGC Method: Improved Corrections for Amines and Amides
Source: Molecules. 2022 Mar 3;27(5):1678. doi: 10.3390/molecules27051678 (PMC8924896; doi:10.3390/molecules27051678)
Supplement: Supplementary file 1 [file molecules-27-01678-s001.zip › molecules-1595931-SI.pdf]

Supplementary Materials:

The PM6-FGC Method: Improved Corrections for Amines and Amides

Martiño Ríos-García, Berta Fernández, Jesús Rodríguez-Otero, Enrique M. Cabaleiro-Lago and Saulo A. Vázquez*

Departamento de Química Física. Universidade de Santiago de Compostela, 15782 Santiago de Compostela (Spain);

* Correspondence: saulo.vazquez@usc.es

Abstract: Recently we reported a new approach to develop pairwise analytical corrections to improve the description of noncovalent interactions by approximate methods of electronic structure, such as semiempirical quantum mechanical (SQM) methods [J. Chem. Theory Comput. 2021, 17, 5556–5567]. In particular, and as a proof-of-concept, we used the PM6 Hamiltonian and we named the method PM6-FGC, where the FGC acronym, corresponding to Functional Group Corrections, emphasizes the idea that the corrections work for specific functional groups rather than for individual atom pairs. The analytical corrections were derived from fits to B3LYP-D3/def2-TZVP (reference) – PM6 interaction energy differences, evaluated for a reduced set of small bimolecular complexes chosen as representatives of saturated hydrocarbons, carboxylic, amine and, tentatively, amide functional groups. For the validation, the method was applied to several complexes of well-known databases, as well as to complexes of diglycine and dialanine, assuming transferability of amine group corrections to amide groups. The PM6-FGC method showed great potential but revealed significant inaccuracies for the description of some interactions involving the –NH_2 group in amines and amides, caused by the inadequate selection of the model compound used to represent these functional groups (an NH_3 molecule). In this work, methylamine and acetamide are used as representatives of amine and amides groups, respectively. This new selection leads to significant improvements in the calculation of noncovalent interactions in the validation set.

Keywords: semiempirical methods, PM6 Hamiltonian, PM6-FGC approach, noncovalent interactions, potential energy curves

Table of Contents

1. Parameters obtained in this work, displayed in the form of a Python dictionary.3

2. **Table S1**. Mean absolute errors (MAEs) calculated for interaction energies below 5, 20 and 40 kJ/mol…………………………………………………………………..7

3. **Figures S1 – S9.** Orientations of the bimolecular complexes considered in the fits and not shown in the manuscript…………………………………………….……8

4. **Figure S10:** Complexes of the S66 database considered for validation …………17

5. **References**……………………………………………………………………….18

Parameters obtained in this work, presented in the form of a Python dictionary.

```
# First group of parameters obtained from best fit for CH3NH2/CH4

C = {('CT','NAN'):(18104.4313178802,2.4311667805,-4256.1629564566,5.8732460758,2.2700807392), \
        ('HCT','NAN'):(5401.8229028156,3.9280582816,-156.365808967,6.8399806694,1.2695536239), \
        ('CT','HNAN'):(-4593.531507152,3.0565546376,-174.4783582368,4.4059369229,1.2), \
        ('HCT','HNAN'):(-119999.6632233276,5.6391276453,699.6309822144,7.0265408068,1.2000003031), \
        ('CAN','CT'):(19224.6993683567,2.5613483969,-3397.8749671693,5.6992899152,2.3878138229), \
        ('CAN','HCT'):(-752.919915889,3.4808504031,-472.709797508,5.1648224459,1.499999808), \
        ('CT','HCAN'):(-69312.1169089271,4.2859628611,3350.9210084273,8.794686474,1.2034609881), \
        ('HCAN','HCT'):(-249999.8409227767,6.0004684057,1054.4316209478,7.5280954438,1.2000000027), \
# Next group obtained from best fit for CH3NH2/CH3NH2
        ('NAN','NAN'):(78296.9416303895,3.0494632988,-1919.5942329666,5.9439554141,2.1027589438), \
        ('HNAN','NAN'):(56653.1711454956,4.5954215756,-1041.3993219922,5.6687988728,1.2623020283), \
        ('CAN','NAN'):(21442.8411958593,3.0752484108,-1297.7803540681,5.6744727564,2.2261683726), \
        ('HCAN','NAN'):(-71121.8449990266,3.9813522942,2975.954674922,6.2578722419,1.5070164559), \
        ('HNAN','HNAN'):(3598.7912860337,2.9754566245,-230.5377350807,6.286007483,1.2), \
        ('CAN','HNAN'):(-15296.9489167049,3.4435238349,214.6076228368,9.0243125585,1.4), \
        ('HCAN','HNAN'):(13772.1689319386,3.8208353133,-347.7391251971,5.7542768688,1.2), \
        ('CAN','CAN'):(61069.7321637047,3.0689345665,-1894.1741049541,5.336222088,2.4308183956), \
        ('CAN','HCAN'):(84697.595281666,6.4069848598,-774.0865573243,6.000241058,1.5370712511), \
        ('HCAN','HCAN'):(-140000.0,5.7882551236,806.2606796849,7.2555538802,1.2), \
# Next group obtained from best fit for CH3NH2/HCOOH
        ('CCA','NAN'):(0.0,4.5970476023,979.2790084852,4.8102878266,2.2591754893), \
        ('NAN','OCA1'):(85731.6336335043,3.1962317098,-1051.320732908,4.7237190674,2.1096525608), \
        ('NAN','OCA2'):(68305.4769265727,3.122415295,194.4474237632,5.8792963777,2.1112896497), \
        ('HOCA','NAN'):(92234.0599076892,4.6853636004,-1283.6039482341,5.449144204,1.1555174846), \
        ('HCCA','NAN'):(10821.8114644893,3.967675857,44.6618825299,7.8428698395,1.4016316028), \
        ('CCA','HNAN'):(-36268.6690455934,4.6264039501,-177.5352419877,5.005079811,1.2850036567), \
        ('HNAN','OCA1'):(-10058.9010874789,5.3922961662,-155.1235434583,7.9980127315,1.2504213283), \
        ('HNAN','OCA2'):(55233.7418329558,5.4633358568,-416.0047121836,6.4160551652,1.2001687284), \
        ('HNAN','HOCA'):(-34500.1299227579,5.0181850203,457.5710023728,6.0,1.2), \
        ('HCCA','HNAN'):(4310.7875007075,5.5333443314,95.8490745437,5.2823760569,1.1765248821), \
        ('CAN','CCA'):(102959.0275505637,4.0647056871,-1839.7255310524,5.2538931659,2.0666759529), \
        ('CAN','OCA1'):(64203.4145035514,3.3299126851,324.7698510893,8.2686818717,2.0350308643), \
        ('CAN','OCA2'):(82181.3882617692,3.2931038195,-608.0710901037,4.9920070863,2.0), \
        ('CAN','HOCA'):(34538.7552499843,4.8602874566,-940.5312888521,5.3695857507,1.4196589055), \
        ('CAN','HCCA'):(13352.1906465115,3.5700312985,-847.5075086884,4.9382253692,1.401967037), \
        ('CCA','HCAN'):(-130000.0,4.9040393436,646.5688819601,6.1328686743,1.3243247507), \
        ('HCAN','OCA1'):(-61310.5747258038,6.5874556811,554.4497223933,6.3953975607,1.5), \
        ('HCAN','OCA2'):(17900.0049011935,3.4558480873,-1376.6675599289,7.2898348277,1.351890849), \
        ('HCAN','HOCA'):(-79514.7875977607,5.6937183752,476.5415535295,7.0026422256,1.1405482908), \
        ('HCAN','HCCA'):(5160.9414623561,5.7700975486,164.6017085442,5.448453096,1.1741621471), \
```

```
# Next group obtained from best fit for CH3CONH2/CH4
    ('CA','CT'):(32866.6253755712,2.8594051823,-2458.2313232663,6.2324031115,2.4665559233), \
    ('CA','HCT'):(-75000.0,4.7871699849,0.0,7.2460451837,1.6043702141), \
    ('CAD','CT'):(41981.8125354367,3.117914468,-1097.6291219146,5.8059393338,2.6), \
    ('CAD','HCT'):(-125000.0,5.5746911841,-2000.0,10.0,1.6), \
    ('CT','NAD'):(15650.7242328132,2.6735025438,-378.4266253352,5.8971324613,2.4000049098), \
    ('HCT','NAD'):(2994.0298993873,2.5240145383,-1261.8403276606,5.8901106019,1.6469340758), \
    ('CT','OAD'):(93237.8775451865,3.3104203821,-695.8693264406,4.7341688695,2.2), \
    ('HCT','OAD'):(10316.3048207605,3.3465826671,-867.7594432849,6.9452724969,1.35), \
    ('CT','HNAD'):(-10113.385879634,3.4223488109,-106.9329111366,6.284711911,1.5), \
    ('HCT','HNAD'):(4794.5355067724,3.567558951,-92.4110150516,5.7120569732,1.1), \
    ('CT','HCA'):(-69953.2993917734,4.7963087118,-146.8727711082,8.0,1.6119651815), \
    ('HCA','HCT'):(6962.5184158169,3.6121714433,-122.9455650428,5.5540472164,1.15), \
# Next group obtained from best fit for CH3CONH2/CH3NH2
    ('CA','NAN'):(24779.6322570372,2.6604411597,-630.1718585948,7.567690749,2.3040810514), \
    ('CA','HNAN'):(-21183.3277080509,3.4939769883,586.5483634756,6.0,1.6019359485), \
    ('CA','CAN'):(59559.1567472948,3.0499133033,-959.5122502983,4.1399352214,2.5201505363), \
    ('CA','HCAN'):(-34778.9272911727,4.3752516699,344.9606761368,7.2963728892,1.7003677445), \
    ('CAD','NAN'):(-118874.6828151514,3.4842540191,1114.8122285086,5.3848509016,2.3837488214), \
    ('CAD','HNAN'):(-48000.0,5.1318080549,-438.285918248,9.4924567836,1.5502997463), \
    ('CAD','CAN'):(68596.3637304978,2.9101081545,-2480.8150832487,4.9700467254,2.6474233143), \
    ('CAD','HCAN'):(12975.6816812637,3.7,-903.4253517177,5.3,1.6102511541), \
    ('NAD','NAN'):(78412.874748562,3.1458770954,902.257479519,4.0483581378,2.2497524365), \
    ('HNAN','NAD'):(24737.9345607368,5.2841255118,-1562.8694813841,6.4415274757,1.4547988002), \
    ('CAN','NAD'):(-12108.2118808438,4.2969919406,1318.3108089656,5.6402834055,2.4282844832), \
    ('HCAN','NAD'):(-77041.9993604651,4.5377262753,1120.4427656906,6.0866451649,1.6055531791), \
    ('NAN','OAD'):(78973.9969615003,3.4265786859,41.0961212004,7.1057099861,2.114667383), \
    ('HNAN','OAD'):(6711.3331166251,5.955816725,-80.0178806452,3.5539595148,1.3), \
    ('CAN','OAD'):(47524.3467301263,3.3154858362,1101.095272475,4.7058403993,2.1), \
    ('HCAN','OAD'):(-3568.4091428034,5.892182651,707.576105386,8.3979924341,1.4751034568), \
    ('HNAD','NAN'):(86831.0907196811,4.5522516121,-1263.6373058661,5.2426595117,1.25), \
    ('HNAD','HNAN'):(25000.0,4.2434695009,-453.7454583873,7.8284628867,1.2), \
    ('CAN','HNAD'):(44050.426349493,3.8057457549,-2108.0447201276,5.4628764931,1.5), \
    ('HCAN','HNAD'):(-63503.254868804,5.3418534481,516.0167890961,6.5556806178,1.1641777844), \
    ('HCA','NAN'):(34502.3940208724,5.1495222232,-238.1936617125,6.2492565057,1.4906248777), \
    ('HCA','HNAN'):(52858.389839301,5.2137965838,-210.1443035681,7.2074047371,1.2404048441), \
    ('CAN','HCA'):(-33078.3155722425,4.1436517214,18.7731517359,7.4125273094,1.6006705289), \
    ('HCA','HCAN'):(1931.4872945196,4.6051949472,214.0661400662,6.2775471214,1.2251918911), \
# Next group obtained from best fit for CH3CONH2/CH3CONH2
    ('CA','CA'):(41120.4360244104,2.7315709733,-3079.9266663575,4.9747921962,2.2880869586), \
    ('CA','CAD'):(20000.0,2.7203077752,-1200.2903663435,6.1898799062,2.4999496914), \
    ('CA','NAD'):(57963.6610936533,3.2713446958,239.2544694434,8.8595141945,2.27), \
    ('CA','OAD'):(135001.1206386854,3.5336540243,-1.4293902668,6.917489556,2.1140194279), \
    ('CA','HNAD'):(-24056.8095848029,3.5957987355,2299.2246324136,9.1701647196,1.4500320757), \
```

```
        ('CA','HCA'):(-65159.5712387253,4.9749797882,-46.560519592,4.9286298262,1.6000921823), \
        ('CAD','CAD'):(-32274.2254647231,4.2369194665,-1115.4733193741,5.9643336089,2.4002772445), \
        ('CAD','NAD'):(-128774.483727511,3.3587232068,-40.4135360013,7.7843288563,2.3432930922), \
        ('CAD','OAD'):(-17903.4405382741,4.8273431756,-499.9639494861,6.2638938591,2.3040805321), \
        ('CAD','HNAD'):(7714.4784039574,2.887319826,-2234.6038491605,7.2171616543,1.5499066606), \
        ('CAD','HCA'):(-59409.8282166785,4.7799399447,-111.1775865842,6.9948270831,1.4085943044), \
        ('NAD','NAD'):(89926.6079847042,2.9313460323,343.0595014166,5.263870236,2.3328693049), \
        ('NAD','OAD'):(117883.690421229,3.3103126179,0.0,5.5170412673,2.2233423521), \
        ('HNAD','NAD'):(-1986.9042699481,2.9000540767,-500.0,4.2473687244,1.3630499974), \
        ('HCA','NAD'):(8537.7998400037,4.9156084603,-305.4687925561,5.6266135532,1.5037594219), \
        ('OAD','OAD'):(129773.6047397633,3.8704842301,197.0448222601,6.0035583444,2.2016569169), \
        ('HNAD','OAD'):(15584.5643393167,3.7900788371,-742.3806742065,5.2189615817,1.2198163413), \
        ('HCA','OAD'):(13225.9692044837,3.9555436451,-378.1953125412,7.1849023483,1.480651083), \
        ('HNAD','HNAD'):(0.0,4.8948063715,130.9004966247,3.6141074021,1.1509816719), \
        ('HCA','HNAD'):(2729.4078013216,3.4688316586,0.8683457291,5.8394475244,1.1004029793), \
        ('HCA','HCA'):(51.4719292782,5.2555153194,196.4395848533,6.3037356816,1.1867045601), \
# Next group obtained from best fit for CH3CONH2/CH3COOH
# CH3-CH3 parameters from CH3CONH2/CH3CONH2
        ('CA','CCA'):(48197.9214321314,3.3592753327,-108.484526595,7.0518104405,2.4637906003), \
        ('CA','OCA2'):(88400.6233673379,3.4143529318,646.978970071,6.1532510659,2.300774825), \
        ('CA','OCA1'):(111140.527749711,3.3851094716,-1554.3911317474,6.0067812799,2.2000972701), \
        ('CA','HOCA'):(4599.0260745488,4.6285308895,-1255.5834393205,6.8533176132,1.4232715483), \
        ('CA','CAD'):(55632.2309295379,3.3325645055,40.7630773886,7.7065286979,2.4981238953), \
        ('CAD','CCA'):(71520.2799505604,4.818445037,-1012.1226259991,5.1464333728,2.4464733431), \
        ('CAD','OCA2'):(-59663.2122843389,3.4920076354,-265.5210630968,8.4344518267,2.4494421066), \
        ('CAD','OCA1'):(-22685.6483382944,4.0838386342,-2101.8328589809,5.4999994818,2.3975183808), \
        ('CAD','HOCA'):(60382.9135888394,4.4544851797,0.0,6.0,1.8497296925), \
        ('CAD','HCA'):(-78231.7617985991,5.2047949427,-45.9291774784,4.522511035,1.6001580078), \
        ('CA','NAD'):(76235.5539834357,3.3074876838,42.3162372738,6.6084453105,2.4256600977), \
        ('CCA','NAD'):(-80706.6554669795,3.5304978042,-651.9088389842,6.7645734115,2.3878519673), \
        ('NAD','OCA2'):(91448.3270845021,3.152184179,-3.3408686294,5.2557037156,2.064124885), \
        ('NAD','OCA1'):(70000.0,3.1898886121,1211.9842531898,4.954901824,2.2), \
        ('HOCA','NAD'):(8991.1112904333,5.2215463043,-741.8235525155,4.9572521753,1.2743303648), \
        ('HCA','NAD'):(-8875.2485153557,5.7499880385,-240.4276336541,5.8787019674,1.6503115002), \
        ('CA','OAD'):(103939.4446795678,3.4714927224,-146.925282775,5.5112760894,2.1019817722), \
        ('CCA','OAD'):(48359.8029918183,4.4892825956,-1294.5093496572,4.9414479661,2.4008618677), \
        ('OAD','OCA2'):(55705.900302597,3.2198319131,-833.082600036,6.4820953956,2.1743288171), \
        ('OAD','OCA1'):(42394.1755514113,4.4829115332,827.5507169976,4.3223539208,2.100521494), \
        ('HOCA','OAD'):(48216.6194287157,4.2164440847,-1068.6776762032,5.3438133348,1.15), \
        ('HCA','OAD'):(7300.4684888114,3.4472779321,-799.7233595994,8.241360388,1.4565817636), \
        ('CA','HNAD'):(-30356.0878609341,5.6750772069,-725.0224127513,5.451046675,1.5624356108), \
        ('CCA','HNAD'):(11140.8766308749,3.3465949634,222.773650818,8.4950579091,1.8997172148), \
        ('HNAD','OCA2'):(26654.4961758333,4.528597037,-619.9371493381,5.8031240772,1.2002994263), \
        ('HNAD','OCA1'):(10164.5950873293,4.0,-400.0,4.5100442362,1.2), \
```

('HNAD','HOCA'):(26740.1608341275,4.6520691907,1.1571204924,8.2294093715,1.2366817144), \
('HCA','HNAD'):(1732.9575770334,3.1291089662,0.0,0.0,1.0885258192), \
('CCA','HCA'):(2040.680141905,4.5598850498,-434.901058802,6.7791363768,1.5512860097), \
('HCA','OCA2'):(6090.6112699615,3.1734725664,-203.4395062544,5.6102250393,1.4506970723), \
('HCA','OCA1'):(4972.4786581267,4.6707473989,910.3519278594,7.5262612757,1.6336519015), \
('HCA','HOCA'):(37274.9342953997,5.4140547197,0.0,0.0,1.1761240484)}

Table S1. Mean absolute errors (MAEs) calculated for interaction energies below 5, 20 and 40 kJ/mol.

		5 kJ/mol	20 kJ/mol	40 kJ/mol
CH_3NH_2 dimer	PM6	1.65	2.69	3.62
	PM6-D3H4	1.32	2.27	3.50
	PM6-FGC	0.34	0.38	0.40
CH_3CONH_2 dimer	PM6	2.51	3.20	4.26
	PM6-D3H4	1.55	2.49	3.86
	PM6-FGC	0.54	0.63	0.76
CH_3NH_2–CH_3CONH_2	PM6	1.80	2.57	3.64
	PM6-D3H4	1.71	2.77	4.40
	PM6-FGC	0.52	0.59	0.68
CH_3NH_2–CH_4	PM6	0.63	1.31	2.76
	PM6-D3H4	0.47	1.15	3.21
	PM6-FGC	0.18	0.26	0.31
CH_3NH_2–HCOOH	PM6	1.76	2.86	4.45
	PM6-D3H4	1.88	3.28	5.23
	PM6-FGC	0.38	0.49	0.57
CH_3CONH_2–CH_4	PM6	0.93	1.71	2.98
	PM6-D3H4	0.61	1.52	3.15
	PM6-FGC	0.15	0.21	0.24
CH_3CONH_2– CH_3COOH	PM6	2.71	3.39	4.67
	PM6-D3H4	1.72	2.90	4.52
	PM6-FGC	0.51	0.68	0.82

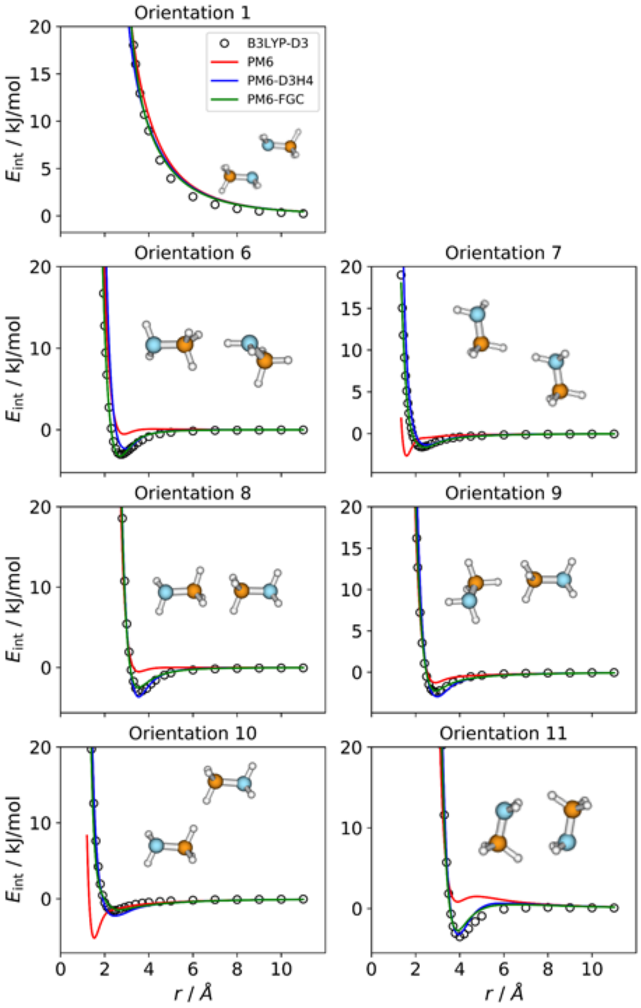

Figure S1. Comparison of IPECs for the orientations of the methylamine dimer not shown in the manuscript.

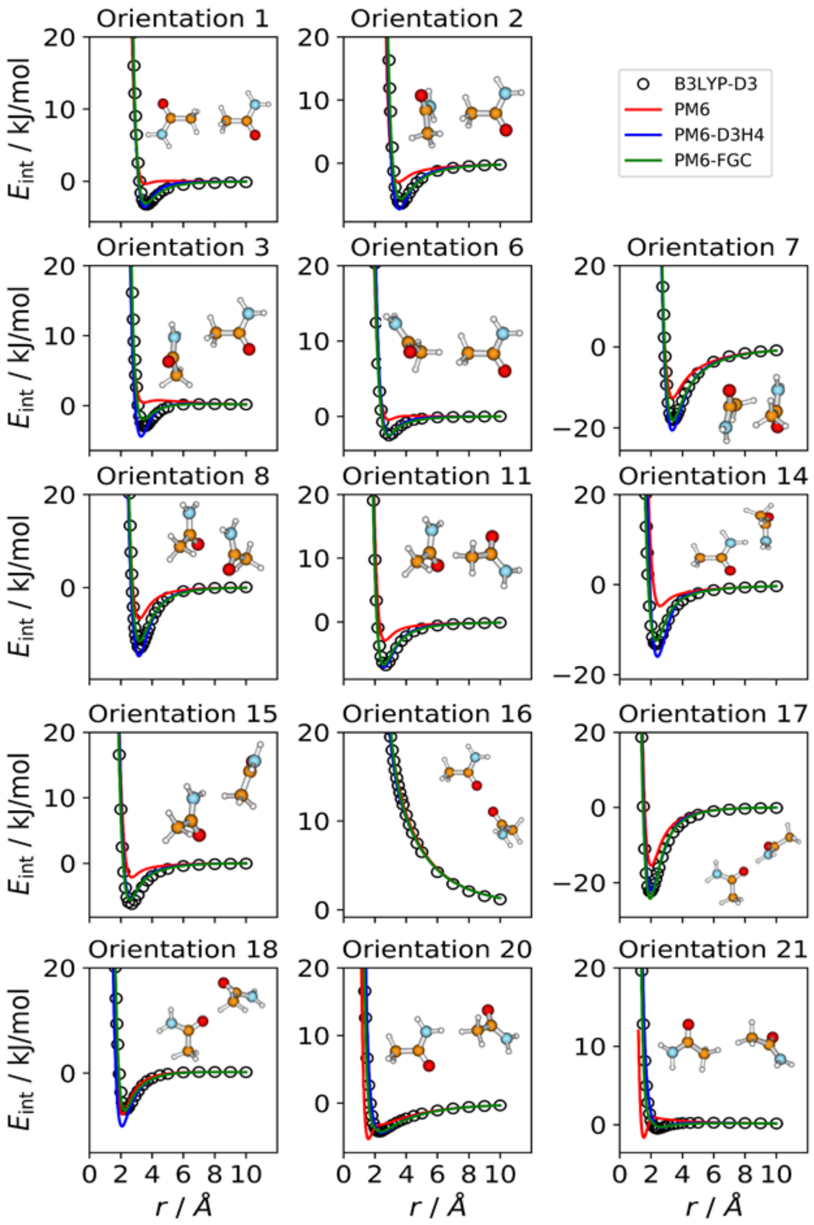

Figure S2. Comparison of IPECs for the orientations of the acetamide dimer not shown in the manuscript.

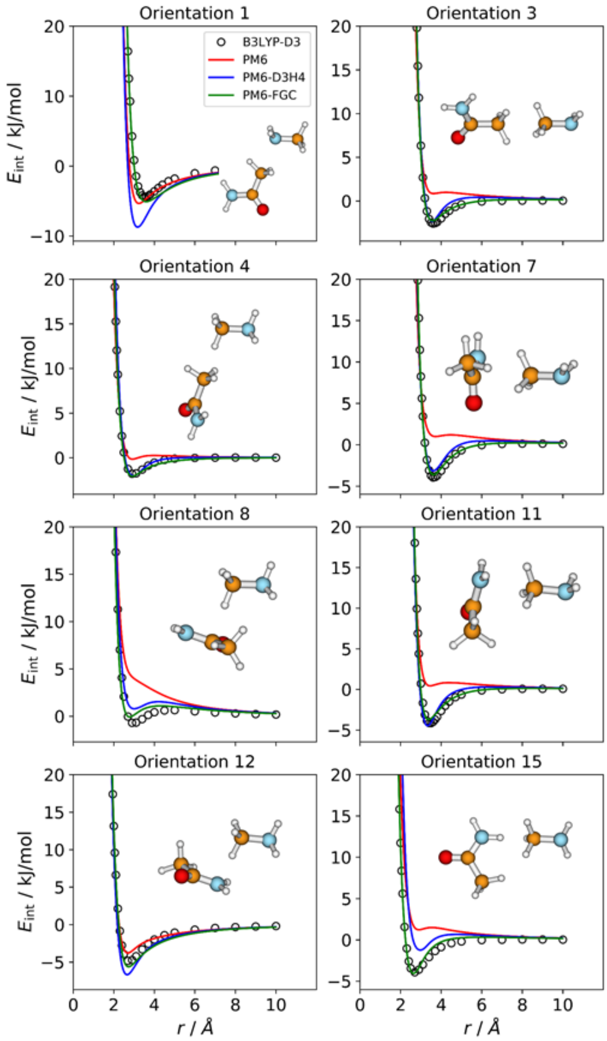

Figure S3. Comparison of IPECs for orientations 1, 3, 4, 7, 8, 11, 12, and 15 of the CH_3NH_2–CH_3CONH_2 complex.

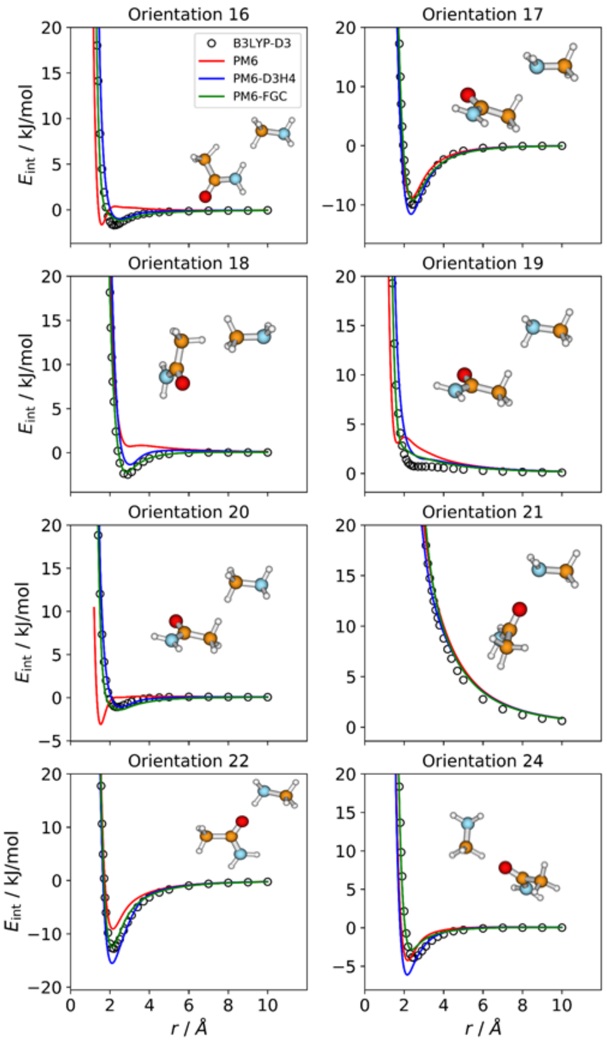

Figure S4. Comparison of IPECs for orientations 16–22, and 24 of the CH_3NH_2–CH_3CONH_2 complex.

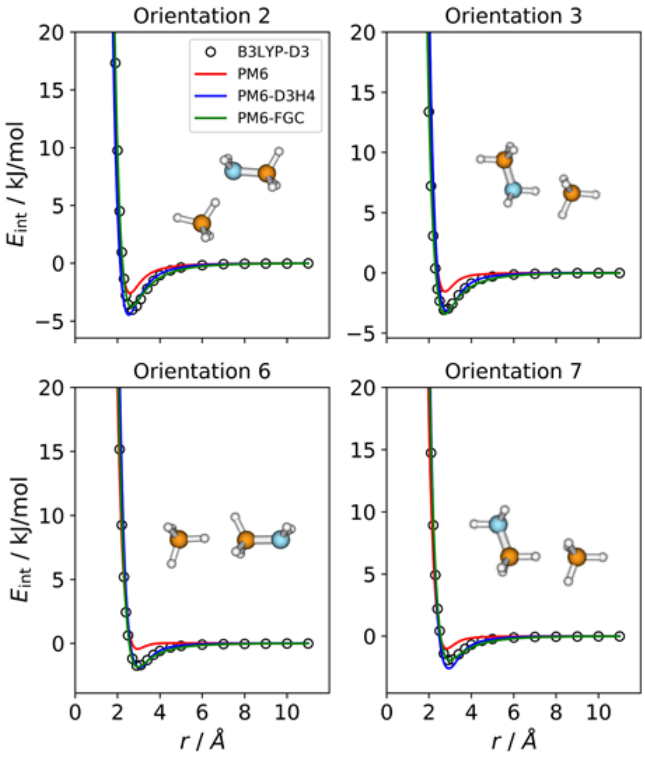

Figure S5. Comparison of IPECs for the orientations of the CH_3NH_2–CH_4 complex not shown in the manuscript.

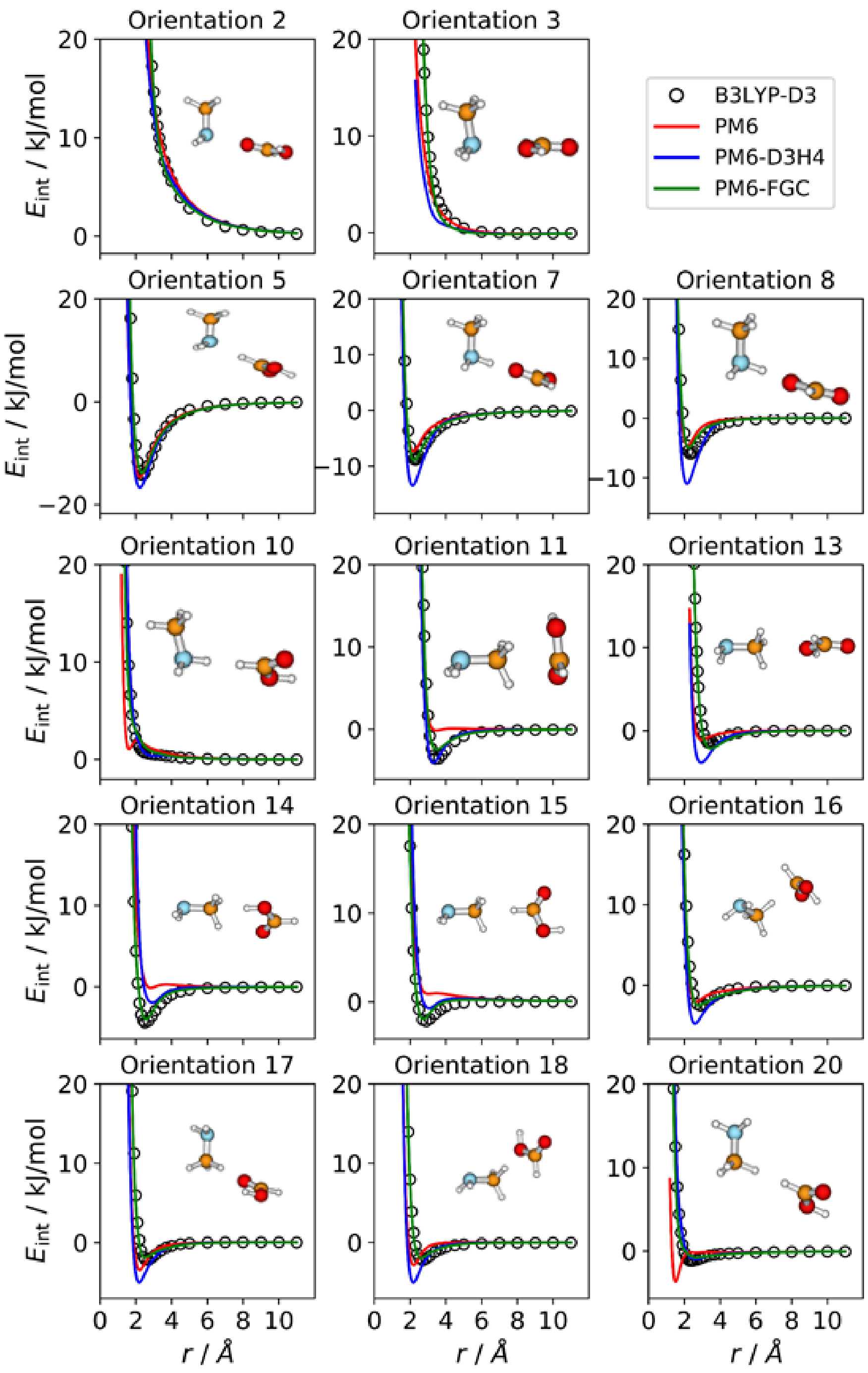

Figure S6. Comparison of IPECs for orientations of the CH_3NH_2–HCOOH complex not shown in the manuscript.

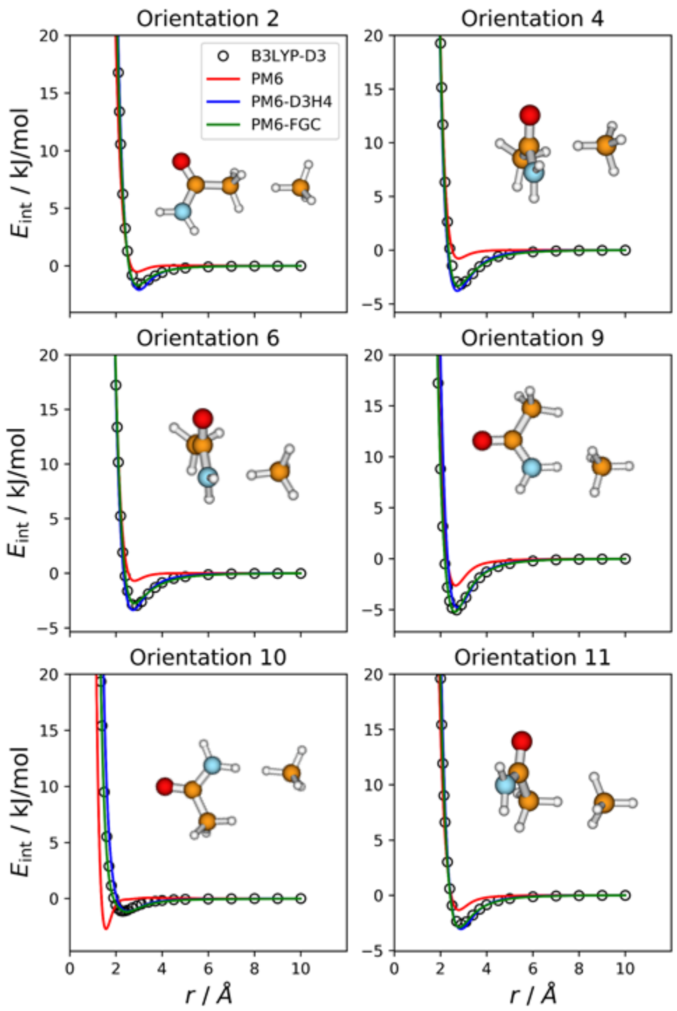

Figure S7. Comparison of IPECs for orientations of the CH_3CONH_2–CH_4 complex not shown in the manuscript.

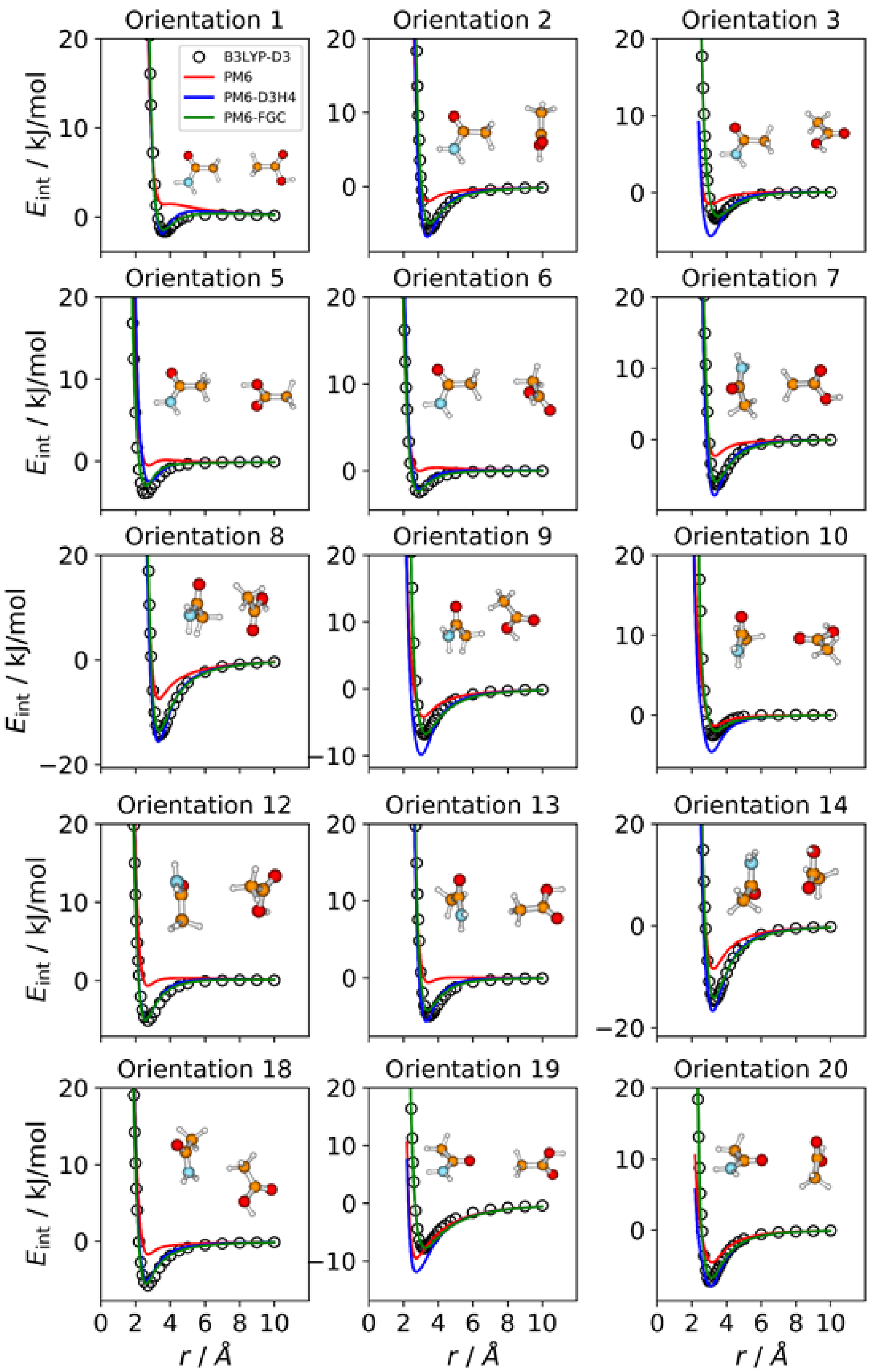

Figure S8. Comparison of IPECs for orientations 1–3, 5–10, 12–14, and 18–20 of the CH_3CONH_2– CH_3COOH complex.

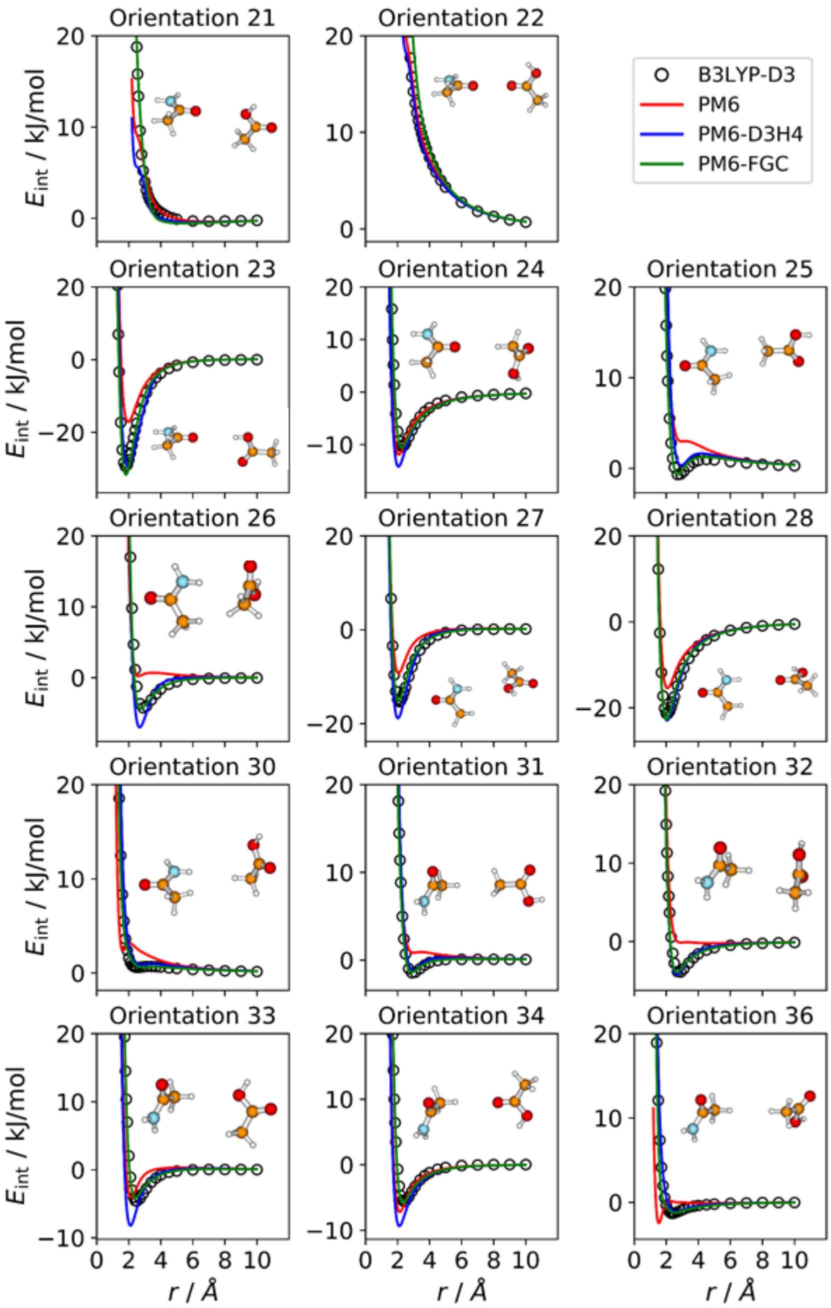

Figure S9. Comparison of IPECs for orientations 21–28, 30–34, and 36 of the CH_3CONH_2–CH_3COOH complex.

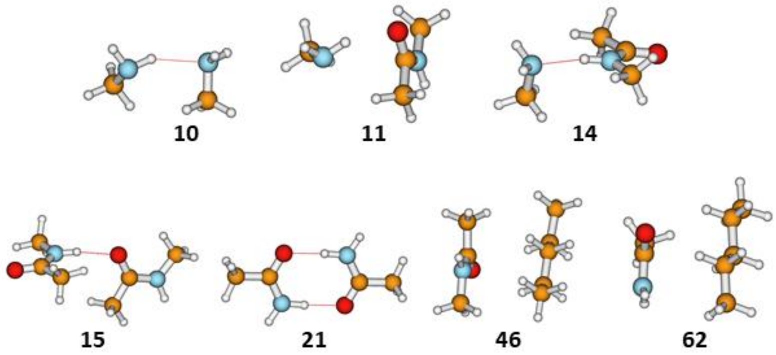

Figure S10. Complexes of the S66 database [1] studied in this work, showing the corresponding identity number.

References

1. Řezáč, J.; Riley, K. E.; Hobza, P., S66: A Well-balanced Database of Benchmark Interaction Energies Relevant to Biomolecular Structures. *J. Chem. Theory Comput.* **2011,** 7, (8), 2427-2438.
